# *In vivo* Activity of Silver Nanoparticles Against *Pseudomonas aeruginosa* Infection in *Galleria mellonella*

**DOI:** 10.3389/fmicb.2020.582107

**Published:** 2020-11-09

**Authors:** Luciana Thomaz, Luiz Gustavo de Almeida, Flávia R. O. Silva, Mauro Cortez, Carlos P. Taborda, Beny Spira

**Affiliations:** ^1^Department of Microbiology, Institute of Biomedical Science, University of São Paulo, São Paulo, Brazil; ^2^Nuclear and Energy Research Institute (IPEN), São Paulo, Brazil; ^3^Department of Parasitology, Institute of Biomedical Science, University of São Paulo, São Paulo, Brazil; ^4^Laboratory of Medical Mycology/LIM53, Faculty of Medicine, Institute of Tropical Medicine of São Paulo, University of São Paulo, São Paulo, Brazil

**Keywords:** *Galleria mellonella*, *Pseudomonas aeruginosa*, silver nanoparticles, hemocytes, kefir

## Abstract

*Pseudomonas aeruginosa* is an opportunistic pathogen associated with life-threatening nosocomial and community-acquired infections. Antibiotic resistance is an immediate threat to public health and demands an urgent action to discovering new antimicrobial agents. One of the best alternatives for pre-clinical tests with animal models is the greater wax moth *Galleria mellonella*. Here, we evaluated the antipseudomonal activity of silver nanoparticles (AgNPs) against *P. aeruginosa* strain UCBPP-PA14 using *G. mellonella larvae*. The AgNPs were synthesized through a non-toxic biogenic process involving microorganism fermentation. The effect of AgNPs was assessed through characterization and quantification of the hemocytic response, nodulation and phenoloxidase cascade. On average, 80% of the *larvae* infected with *P. aeruginosa* and prophylactically treated with nanoparticles survived. Both the specific and total *larvae* hemocyte counts were restored in the treated group. In addition, the nodulation process and the phenoloxidase cascade were less exacerbated when the *larvae* were exposed to the silver nanoparticles. AgNPs protect the *larvae* from *P. aeruginosa* infection by directly killing the bacteria and indirectly by preventing an exacerbated immunological response against the pathogen. Our results suggest that the prophylactic use of AgNPs has a strong protective activity against *P. aeruginosa* infection.

## Introduction

*Pseudomonas aeruginosa* is a γ-proteobacterium found in natural reservoirs of water, soil, plants and animals, being also common in hospital premises. This bacterium has the ability to colonize surfaces and medical equipment, frequently leading to nosocomial infections (Decraene et al., 2018). In humans, *P. aeruginosa* infections are associated with severe skin burns and is a major cause of death in patients with cystic fibrosis (Emerson et al., 2002; Rodríguez-Rojas et al., 2012). *P. aeruginosa* is classified as a serious health threat by the Center for Disease Control and Prevention (CDC) and due to its drug resistant nature is considered a Priority 1 critical pathogen by the World Health Organization (WHO) (Magiorakos et al., 2012). Most clinical strains of *P. aeruginosa* display resistance to several classes of antibiotics such as β-lactams, carbapenems, aminoglycosides, and fluoroquinolones (Potron et al., 2015). Colistin is a last- resort antibiotic used in *P. aeruginosa* therapy, but it displays nephro and neurotoxicity. Furthermore, strains of *P. aeruginosa* resistant to colistin have already been reported (Hill et al., 2014; Karaiskos et al., 2017). Hence, there is an urgent need for the screening and development of new antimicrobial drugs effective against *P. aeruginosa*. Silver (Ag) displays antibacterial activity and has been widely used throughout history in the fight against bacterial infections (Fox, 1968; Mathur et al., 2018). Silver-containing products have been used in medical devices to prevent contamination with opportunistic pathogens such as *Candida* and *Aspergillus* (Jain et al., 2009). The use of silver in anti-*P. aeruginosa* therapy began in 1968 with the introduction of silver nitrate for the prophylactic treatment of burn wounds (Fox, 1968). More recently, silver nanoparticles (AgNPs) have been devised to improve the physical, chemical, and biological properties of Ag. AgNPs display a strong antimicrobial activity even against bacteria resistant to multiple antibiotics, making it an alternative weapon in the fight against pathogenic microorganisms (Kalishwaralal et al., 2010; Rai et al., 2012; Mathur et al., 2018). Although the bactericidal properties of AgNPs have been demonstrated (Sondi and Salopek-Sondi, 2004; Markowska et al., 2013; Yan et al., 2018), the exact mechanism of action of these nanoparticles is still unclear. It has been suggested that AgNPs directly interact with the bacterial cell wall or that they release Ag^+^ ions which in turn exert toxicity on the pathogen (Yan et al., 2018). AgNP causes the denaturation of the 30S ribosome subunit, impairing the expression of essential proteins (Rai et al., 2012). It also binds to RNA and DNA molecules leading to the formation of nucleotide dimers (Sondi and Salopek-Sondi, 2004). On another level, AgNPs strongly bind membranal lipids, where they poke holes and affect the bacterial wall structure resulting in the leak of the intracellular content (Rai et al., 2012). AgNPs also interfere with cellular signaling by dephosphorylating tyrosine residues on key bacterial peptide substrates and ultimately inhibiting microbial growth (Shrivastava et al., 2007). Cellular oxidative stress is an indication of toxic effects caused by heavy metals ions, such as Ag^+^. At least part of the potent antibacterial, antifungal and antiviral activity of AgNPs is due to their ability to produce radical oxygen species (ROS) such as hydrogen peroxide (H_2_O_2_), superoxide anion (O_2_^–^), hydroxyl radical (OH·), hypochlorous acid (HOCl) and singlet oxygen (Wu et al., 2014).

A critical aspect in the evaluation of an antimicrobial drug is the pre-clinical test for effectiveness and toxicity in an animal model. Although rodents are the most common model for the investigation of pathogen-host interactions, they are costly and require a relatively complex infrastructure. A viable alternative is the use of invertebrates that in addition of supporting the 3R principle (reduction, refinement, and replacement) are also exempt from ethics committee approval. The greater wax moth, *Galleria mellonella*, exhibits specific characteristics that make it suitable for the study of host-pathogen interactions. This invertebrate has many advantages over other animal models (reviewed by Cutuli et al., 2019) and is becoming a major model of infection by pathogenic microorganisms. Their *larvae* can be incubated at 37℃, and they display an innate immune response similar to that of vertebrates, which are in many aspects conserved in metazoans (Browne and Kavanagh, 2013; Wojda, 2017). Thus, *G. mellonella* is a simple, inexpensive and fast-testing *in vivo* model for studying microbial virulence and for screening new antimicrobial agents (Desbois and Coote, 2012; Hill et al., 2014). Indeed, several studies have already explored *G. mellonella larvae* as a model for *P. aeruginosa* infection (Miyata et al., 2003; Andrejko et al., 2014; Hill et al., 2014; Mizerska-Dudka and Andrejko, 2014; Beeton et al., 2015).

In the present study, we assessed the antibacterial activity of a new AgNP formulation against *P. aeruginosa* strain UCBPP-PA14, using *G. mellonella larvae* as an infection model. This is a highly virulent strain in both animals and plants which carries two pathogenicity islands (He et al., 2004; Lee et al., 2006) and that is gradually substituting strain PA01 in *P. aeruginosa* pathogenicity studies (Mathee, 2018). A thorough analysis of the *larvae* immune response was conducted and a mechanism for the antipseudomonal activity of biogenic AgNPs is suggested.

## Materials and Methods

### Insects and Microorganisms

The *larvae* of the greater wax moth *Galleria mellonella* (order Lepidoptera, family Pyralidae and subfamily Gallerinnae) were reared on artificial diet (pollen and honeybee manufactured by Apiário Seiva das Flores/São Paulo, Brazil) at 28.5℃ in our animal facility. *G. mellonella larvae* in the final (7th) larval instar and weighing 150–220 mg were used in all experiments. Only *larvae* with intense movement and devoid of black spots in the cuticle were used. The colonies were routinely tested for the presence of pathogens, such as filamentous fungi. Although there are not formal ethical regulations on experimental work with *G. mellonella*, the ethical principles of reduction, refinement and replacement have been implemented throughout this study. The insects were sacrificed by freezing. It has been shown that food deprivation leads to increased infection susceptibility, due to the suppression of immune responses (Banville et al., 2012). To avoid this additional confounding effect, *larvae* were fed *ad libitum* for the duration of the study.

*P. aeruginosa* strain UCBPP-PA14 was routinely grown at 37℃ in Lysogeny Broth (LB)/L-agar medium (Miller, 1992) or in Mueller-Hinton medium.

### Synthesis and Characterization of AgNP

AgNPs were synthesized in the Biomaterials Laboratory, at the Science and Technology Material Center (CCTM) of the Nuclear and Energy Research Institute (IPEN) in São Paulo, Brazil. The green synthesis of silver nanoparticles was performed using a water kefir liquor, which is produced through the fermentation of a raw sugar solution containing kefir grains (consisting of polysaccharides and microorganisms). Kefir liquor contains ethanol, glycerol, lactic acid, acetic acid, mannitol, ethyl acetate, isoamyl acetate, ethyl hexanoate, ethyl octanoate, and ethyl decanoate (Laureys and De Vuyst, 2014). This is to the best of our knowledge the first time that fermenting kefir liquor has been used for the green synthesis of silver nanoparticles. The kefir grains were gently stirred (RW 20 digital, IKA, Germany) in deionized water for 1 h and sieved. This washing process was repeated thrice. The kefir fermentation was performed at room temperature, for 48 h, where 200 g of kefir grains and 50 g of organic unrefined sugar cane were mixed in 500 ml deionized water. The water kefir fermented liquor was centrifuged (16,000 × g, 15 min, 4℃) to obtain a cell-free supernatant (Solution A). For the green synthesis of silver nanoparticles, 10 ml of Solution A were mixed with 0.01 g of AgNO_3_ (Sigma Aldrich), under ultrasonic agitation for 10 min. The AgNPs were stored under refrigeration (4℃) and protected from light until further use. The nanoparticles were characterized by transmission electron microscopy (TEM) (JEOL JEM 2100) at an accelerating voltage of 200 kV and UV—Visible spectroscopy (Epoch 2 Biotech).

### Determination of AgNP Minimum Inhibitory Concentration (MIC)

An inoculum of 10^6^ bacteria/ml was suspended in Mueller-Hinton medium containing increasing concentrations of silver nanoparticles (0–10 μg/ml) and grown for 24 h at 37℃. The MIC was determined by measuring the optical density of the cultures at 600 nm (Andrejko et al., 2014). Experiments were performed three times independently.

### Toxicity of AgNPs in *G. mellonella*

The toxicity assay was performed *in vivo* using *G. mellonella larvae*. Groups of 18 *larvae* were treated with one of the following concentrations of AgNPs: 25, 35, 50, 75, 85, and 100 mg/K and *larvae* survival was monitored for 8 days. *Larvae* that presented lack of movement and did not respond to touch were considered dead. Experiments were performed three times independently. The Spearman–Karber Method was used to estimate the Lethal Dose causing 50% mortality (LD_50_) of *G. mellonella larvae*.

### Prophylactic Treatment

*Larvae* were treated with either 5 μl of 0.9% NaCl, Solution A, AgNP (5–25 mg/Kg) or AgNO_3_ (25 mg/Kg) by injection into the haemocoel of the last right pro-leg, using a 10-μl Hamilton microsyringe. The *larvae* were kept in Petri dishes and incubated at 37℃, for 2 h before bacterial infection.

### Infection of *larvae*

The external body of the *larvae* was sterilized with 70% ethanol. For the infection, 100 bacteria/10 μl were injected intra-haemocoel in the last left proleg, and the *larvae* were incubated at 37℃ in the dark. Higher doses of bacteria (>100 CFU) killed all *larvae* in less than 24 h (not shown). Three negative control groups—*larvae* inoculated with either 0.9% NaCl, or Solution A, or an untouched group in which the *larvae* were only rinsed with ethanol, were included in all experiments. The latter control group was omitted from the figures to avoid cluttering, but in all cases it showed essentially the same results as the 0.9% NaCl or Solution A controls. The *larvae* were visually monitored for 4 consecutive days. Death was determined by the lack of movement in response to touch and by dark pigmentation on the *larvae* cuticle. Each treatment group contained at least 20 *larvae* and all experiments were performed at least three times.

### *Galleria mellonella* Survival Assay

*Larvae* mortality was assessed by determining the percentage of survival over 4 days using a Kaplan–Meier plot and the Log-rank test (GraphPad Prism 6.0.1, GraphPad Software, Inc., La Jolla, USA). *larvae* that did not respond to physical touch and showed strong pigmentation on the cuticle were considered dead. Each group consisted of 20 *larvae*, and each survival assay was performed three times, totalizing 60 *larvae* per group in total.

### Extraction of Hemolymph

The *larvae* were punctured in the abdomen with the help of a sterile scissor. The hemolymph was collected in microtubes containing Insect Physiological Saline (IPS—150 mM NaCl, 5 mM KCl, 0.1 M Tris-HCl, pH 6.9) and 0.002% phenylthiourea (PTU) (Sigma-Aldrich). Samples used to assess ProPO activity were extracted in the absence of PTU, and all samples were kept on ice to prevent coagulation/melanization. Cells were then centrifuged for 5 min at 200 × g at 4℃. The pellet was suspended in IPS and immediately used for hemocyte counting and to determine bacterial concentration (CFU/ml).

### Determination of Bacterial Load in *G. mellonella*

*larvae* were bled 3, 6, 9, or 18 h following infection. Hemolymph was obtained as described above and plated on L-agar containing 25 μg ml^-1^ nalidixic acid. Plates were incubated overnight at 37℃ and colonies were counted. This assay was performed three times with 16 *larvae* for each condition.

### Quantification of Hemocytes

The number of hemocytes in *G. mellonella* was determined through total hemocyte count (THC), and differential hemocyte count (DHC). Hemocytes were collected at 3, 6, 9, and 18 h after infection and counted using a phase contrast microscope. Cell types were classified as follows: plasmatocytes (Pl), granular cells (Gr) oenocytoids (Oe), or spherulocytes (Sp). Pl and Gr were identified by the presence of small granules in both population types and an extensive cytoplasm spread (exhibited only by Pl). Oe were identified by the presence of big cell bodies and big nuclei. Sp cells were identified by the presence of spherules, as described (Andrejko et al., 2014; Mizerska-Dudka and Andrejko, 2014; Wu et al., 2016). These experiments were performed three times with 16 *larvae* for each condition.

### Phenoloxidase Activity

The *larvae* hemolymph was extracted and suspended in ice-cold IPS, without PTU. Two microliters of the suspension were diluted in 18 μl Tris buffer saline (TBS) containing 5 mM CaCl2 and 2 mM L-DOPA (Sigma - Germany), dissolved in 50 mM sodium phosphate pH 6.5. Melanin formation was quantified by reading the absorbance at 490 nm in a spectrophotometer (Epoch 2 Biotech) over 90 min, at 15 min intervals. Each assay was performed three times, with 16 *larvae* for each condition.

### Hemocyte Aggregation Assay (Nodulation Assay)

Hemocytes were collected from non-infected and infected *larvae* and sampled on coverslips placed inside a 24 cell culture plate containing RPMI 1640, 10% FBS and 1% penicillin/streptomycin, and incubated at 30℃ in a CO_2_ chamber. After 3 h, the samples were washed with IPS. The hemocyte aggregates were counted in a DMI6000B/AF6000 microscope coupled to a DFC365FX camera with the help of the LAS software for image capture (Leica).

### Determination of Bacteria-Hemocytes Interaction

The interaction between *P. aeruginosa* and hemocytes was assessed *ex vivo* and by immunofluorescence. Hemocytes were collected from non-infected *larvae*, suspended on a 24-cell culture plate filled with RPMI 1640, 10% FBS and 1% penicillin/streptomycin and incubated at 30℃ in a CO_2_ chamber at 5% CO_2_ for 1 h to allow the attachment of the hemocytes to the well surface. Approximately 1,000 TAMRA (tetramethylrodhamine, 5 μM)-stained bacteria were then added to the wells, each containing approximately 10^6^ hemocytes. After 3 h the samples were washed with IPS containing 100 μg/ml gentamicin to remove non-internalized bacteria. The hemocytes were then fixed with 2% paraformaldehyde for 10 min, washed with TBS, and incubated for 15 min in 50 mM ammonium chloride. The samples were then washed with TBS, and the cells were permeabilized with a solution containing 0.1% saponin, 0.1% sodium azide and 1% BSA. Next, the samples were stained with phalloidin-Alexa 488 (Thermo) for 2 h at 8℃, washed with TBS and mounted on a slide with the help of Prolong-Diamond Antifade Mountant (Thermo). For the Live imaging time-lapse video, TAMRA-loaded bacteria were washed several times to discard any extracellular fluorescent marker and incubated with hemocytes previously plated in a MatTek 35 mm petri dish containing a 1.5 mm coverglass (MatTek Corporation, Ashland, MA, USA) and processed for the capturing of the z section. Fixed samples or Petri dishes were visualized on a fluorescence microscope (Leica DMI6000B/AF6000) coupled to a digital camera system (DFC 365 FX) by using Leica Application Suite X (LAS X) and Image J software (Schneider et al., 2012).

### Statistical Analyses

All experiments were analyzed using GraphPad Prism 6.0.1 The statistical analyses were performed by ANOVA and RM ANOVA followed by *t*-test and the survival assays were analyzed by the Kaplan–Meier Curves and Mantel–Cox tests.

## Results

### Characterization of AgNPs

**Figure 1:**
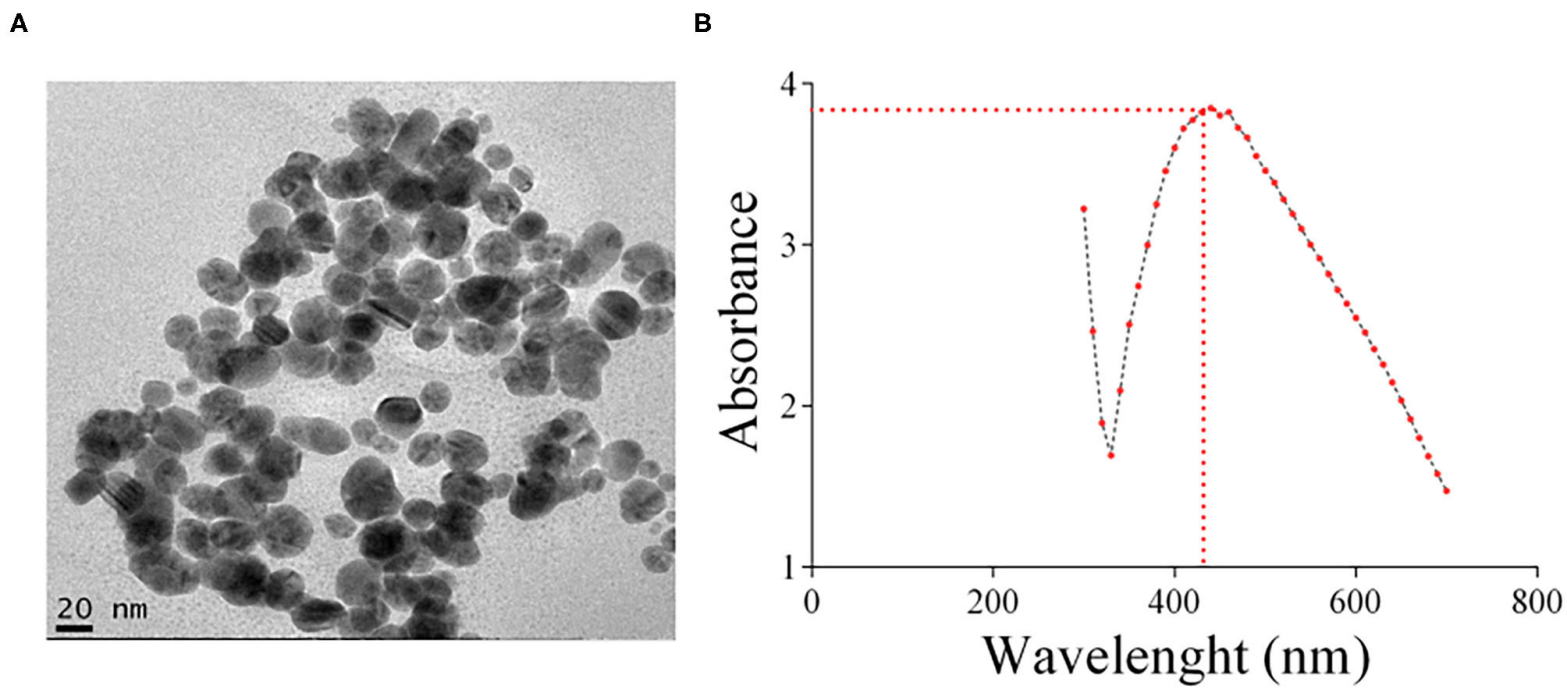
**(A)** Representative TEM image of the AgNPs prepared and used in this study. **(B)** UV/VIS absorption spectra of the silver colloid nanoparticles. The absorbance peak of the AgNPs corresponding to the surface plasmon resonance (SPR) of the nanoparticles was 435 nm.

AgNPs were synthesized by a biogenic process that uses kefir as described in Materials and Methods and characterized by TEM and UV/VIS absorption spectra (Figure 1). The diameter of a typical AgNP was about 20 nm. For two months, once a week, the stability of the silver nanoparticles was monitored. AgNPs were highly stable, alterations in color or visual aggregation were never observed. The absorbance peak of the AgNPs was at 435 nm (Figure 1B), which corresponds to the surface plasmon resonance (SPR) of the nanoparticles. It is worth noticing that the process used for AgNP synthesis is non-toxic and inexpensive, being an alternative to physico-chemical methods that are costly and potentially toxic (Singh et al., 2015).

### Minimal Inhibitory Concentration of AgNP

AgNP antibacterial activity against *P. aeruginosa* (strain PA14) was evaluated in a MIC assay *in vitro*. PA14 is a highly virulent *P. aeruginosa* clinical isolate. This strain is a representative of the most common clonal group of this bacterial species (Wiehlmann et al., 2007). Increasing concentrations of AgNPs were added to a PA14 culture in MH medium that was subsequently grown for 24 h. The MIC of our AgNPs was 2 μg/ml, slightly higher than the 1.06 μg/ml reported by Pompilio et al. (2018) (*P. aeruginosa* strains PA14 and AC12A) but significantly lower than the MIC of AgNPs on *P. aeruginosa* reported by others: 8 μg/mL (strainPA01) (Radzig et al., 2013), 16 μg /mL (Wypij et al., 2018) (strain ATCC10145) and 14 μg/mL – 29 μ g /mL (strain CCM3955) (Guzman et al., 2012), 2.48 μ g/mL (clinical isolates) (Liao et al., 2019).

### Toxicity of AgNPs in *G. mellonella larvae*

**Figure 2:**
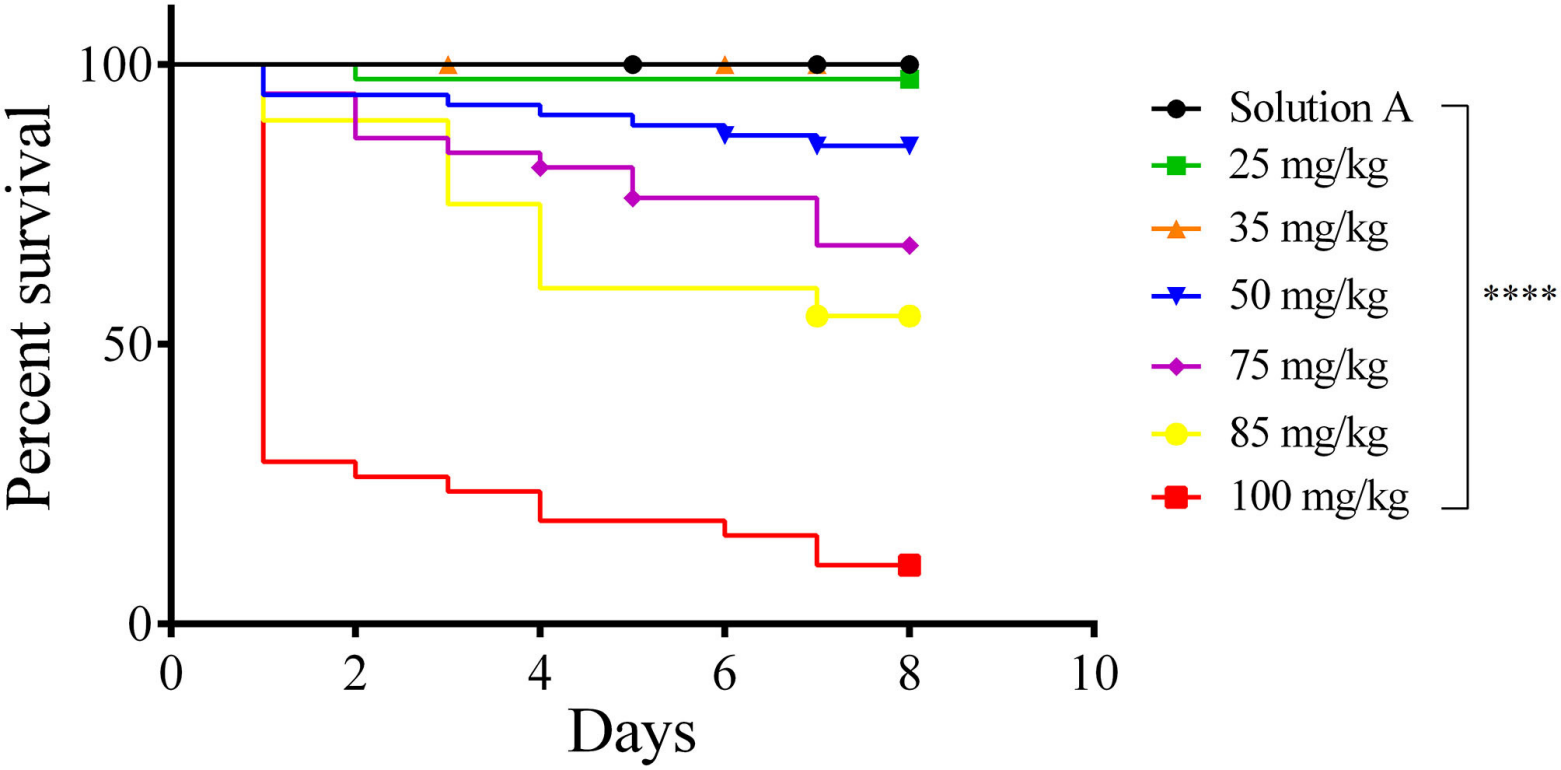
Survival curve (Kaplan–Meier and Mantel Cox). Toxicity of AgNP in *Galleria mellonella*. Groups of 18 larvae each were treated with 25–100 mg/Kg of *larvae* tissue. The control groups were Solution A (black line) and Sham (not shown). *Larvae* were incubated at 37℃ and their viability was followed for 8 days. Each line represents the mean of three independent experiments. ^****^p < 0.0001.

To test whether our AgNP preparation bears toxicity against *G. mellonella larvae*, a toxicity assay with increasing concentrations of AgNPs (25, 35, 50, 75, 85, and 100 mg/Kg of *larvae*) was conducted (Figure 2). *Larvae* viability was observed for 8 consecutive days. No toxicity was observed at concentrations below 35 mg/Kg. The LD_50_ of this AgNP formulation is 68.70 mg/Kg of *larvae*.

### Protective Effect of AgNP on *G. mellonella* Infected With *P. aeruginosa*

**Figure 3:**
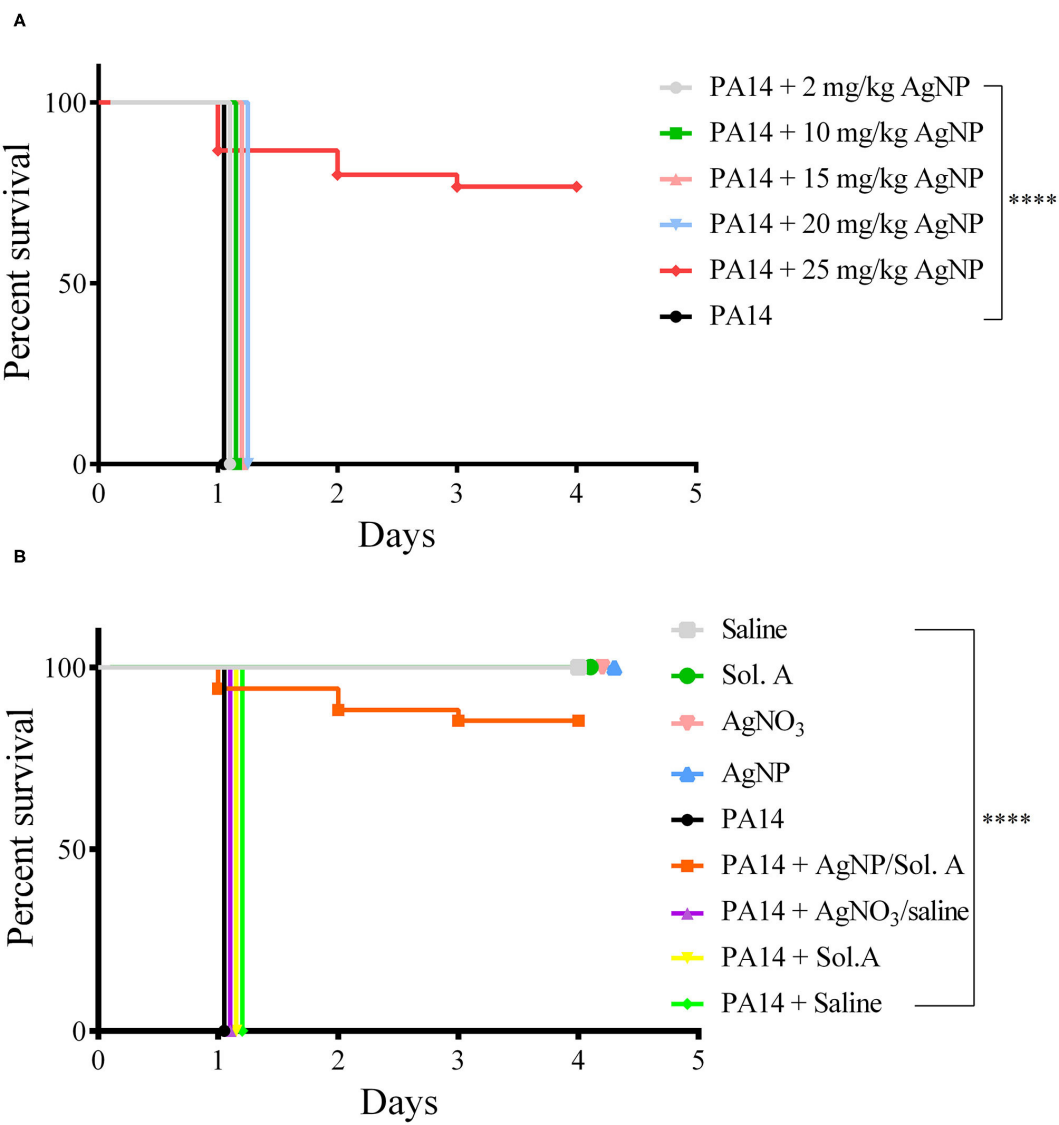
Survival curves (Kaplan–Meier and Mantel Cox). **(A)** Protective effect of AgNPs in *G. mellonella* infected with *P. aeruginosa*. Groups of 20 *larvae* each were treated prophylactically for 2 h before infection, with 5–25 mg/Kg AgNPs per larva. The *larvae* were then infected with 100 CFU of *P. aeruginosa* strain PA14. The black line represents *larvae* infected with PA14 in the absence of any prophylactic treatment. **(B)** Groups of 20 *larvae* were prophylactically treated with 25 mg/Kg AgNPs, Solution A, Saline or AgNO_3_. Two hours later, they were infected with 100 *P. aeruginosa* cells. The control groups were: Saline (gray line); Sol. A (green); AgNO_3_ (pink); AgNPs (blue). All *larvae* groups in A and B were incubated at 37℃ and their viability was followed for 4 days. Each line represents the mean of three independent experiments. ^****^p < 0.0001.

A preliminary assay showed that a concentration of 25 mg/Kg AgNPs was required to confer on the *larvae* protection against *P. aeruginosa* (*p* = 0.0001, Logrank (Mantel–Cox) and Kaplan–Meier tests), while lower concentrations—5–20 mg/Kg—were not sufficient to kill the bacteria (Figure 3A). In addition, no toxic effect against *G. mellonella* could be observed at this concentration. Therefore, 25 mg/Kg was chosen as the working concentration of AgNPs. A thorough examination of the prophylactic effect of AgNPs or AgNO_3_ followed (Figure 3B). A group of *larvae* was inoculated with silver nanoparticles 2 h prior to bacterial infection. At time 0 h, the *larvae* were infected with 100 *P. aeruginosa* cells. *larvae* that were not exposed to the prophylactic AgNPs treatment (group 1 - PA14, group 2 - PA14 + Sol. A, group 3 - PA14 + 0,9% NaCl and group 4 - PA14 + AgNO_3_/saline) exhibited disease symptoms (slow movement, loss of turgor and body darkening), and died during the first 24 h following infection. Eighty percent of the *larvae* pre-treated with 25 mg/Kg AgNPs and then challenged with *P. aeruginosa* were still alive 4 days after infection (*p* = 0.0001, Log-Rank - Mantel Cox and Kaplan–Meier test). Interestingly, 25 mg/Kg AgNO_3_ did not confer protection on the *larvae* infected with *P. aeruginosa*.

### *P. aeruginosa* Growth Inside *G. mellonella*

**Figure 4:**
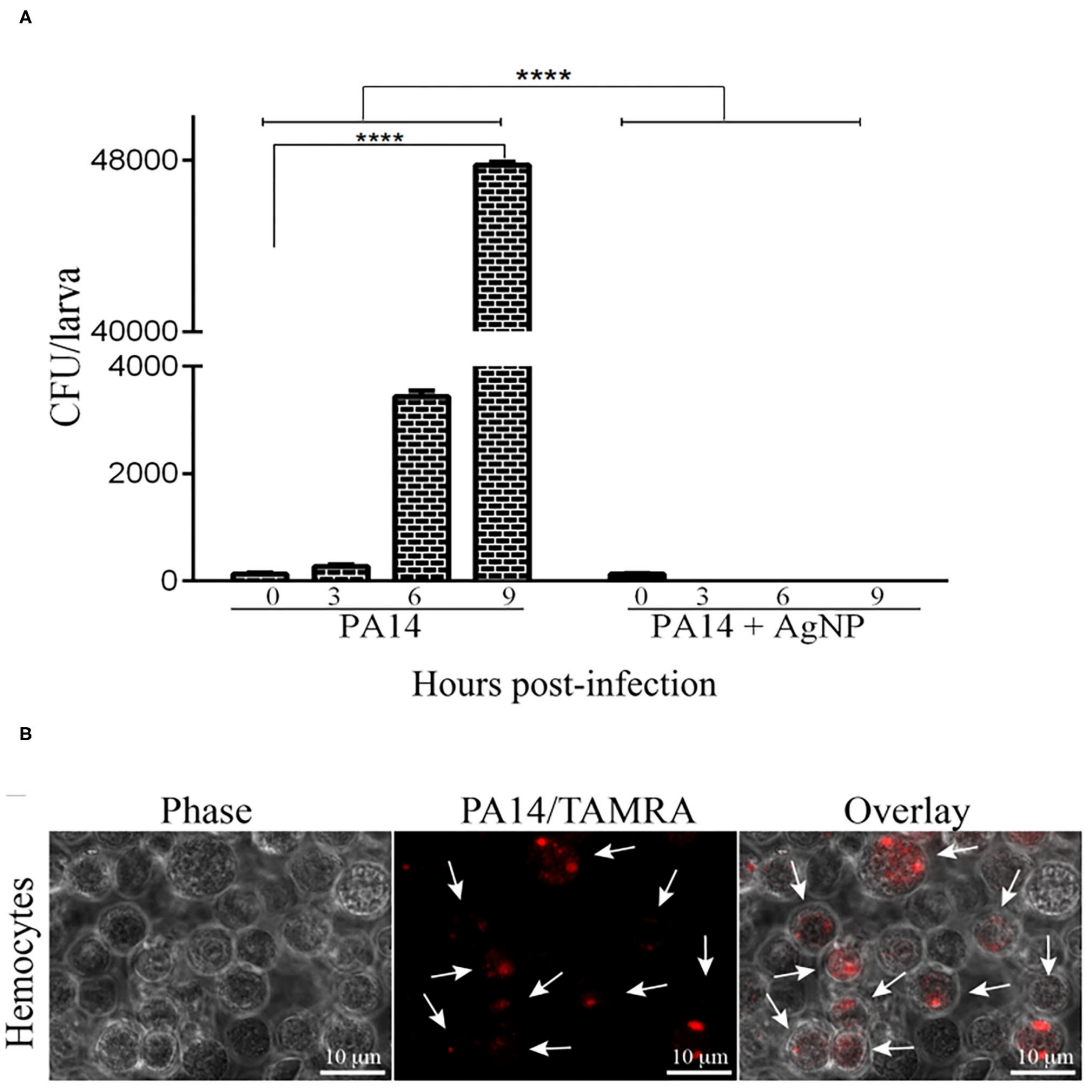
Bacterial growth in the haemolymph of infected *larvae*. **(A)** 16 *larvae* were pre-treated with AgNPs and inoculated with *P. aeruginosa*. Zero, three, six, and nine hours following infection, the *larvae* haemolymph was extracted and plated on L-agar plates containing 25 μ g/ml nalidixic acid. Following a 24 h incubation at 37℃ the CFU on the plates were counted. Each bar represents the mean of three independent experiments. ^****^p < 0.0001 (ANOVA and RM ANOVA followed by *t*-test). **(B)** Microscopic images of hemocytes from *G. mellonella* internalized with PA14. Bacteria cells were stained with TAMRA. Scale bars 10μ m. White arrowheads point to bacteria inside the hemocytes. Photos are representative of three independent experiments.

The progress of infection was followed by counting *P. aeruginosa* CFU in the hemolymph of the infected *larvae*. Figure 4A shows that in the group pre-treated with AgNPs no bacteria were observed at any time (*p* < 0.0001; Two-Way ANOVA followed by *t*-test). However, in the *larvae* that did not receive the prophylactic treatment there was a steady increase in bacterial concentration (*p* < 0.0001; Two-Way ANOVA followed by *t*-test). Figure 4B shows the interaction between bacteria stained with TAMRA supravital staining (PA14/TAMRA) and hemocytes. It can be observed that the hemocytes internalized the bacteria, which kept growing inside the hemolymph. The presence of bacteria inside the cells was confirmed by a z section of an infected cell sample (see video in the Supplementary Material).

### Hemocyte Quantification and Characterization

**Figure 5:**
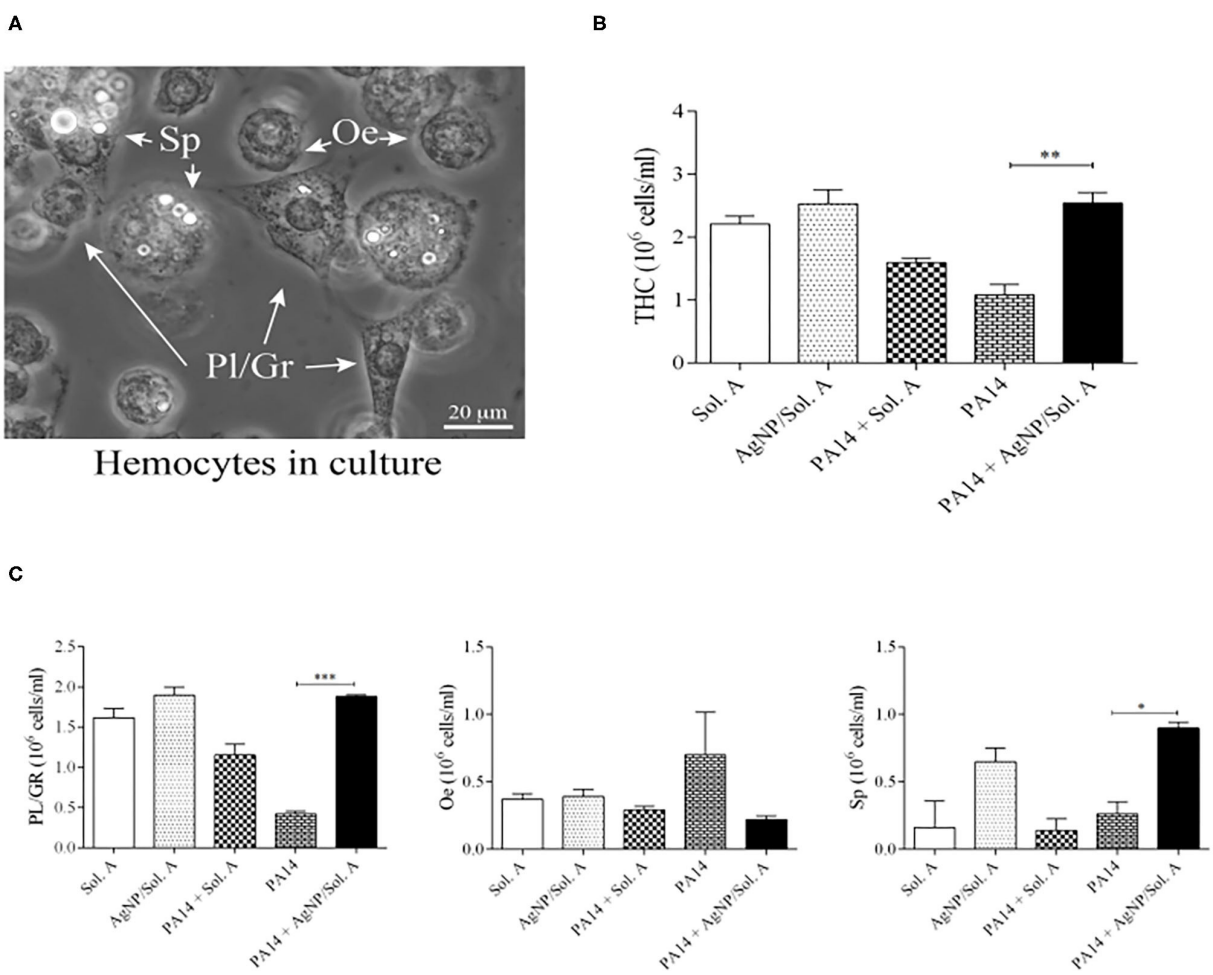
**(A)** Microscopic images of *Galleria mellonella* hemocytes. Bar = 20 μ m. White arrowheads point to hemocytes, Pl/Gr, plasmatocytes/granular cells; Sp, spherulocytes, Oe, oenocytoids. **(B)** Total Hemocyte Count (THC) and **(C)** Differential Hemocyte Count (DHC) of circulating hemocytes in *larvae* prophylactically treated for 2 h with Solution A or AgNP/Sol. A and then infected for 18 h with *P. aeruginosa* cells. The first and second bars represent *larvae* treated with Sol. A and AgNP/Sol. A, respectively (negative controls). The third bar represents *larvae* infected with bacteria (PA14) in the presence of Sol. A. The fourth bar corresponds to *larvae* infected with bacteria suspended in saline and the fifth bar corresponds to *larvae* pre-treated with AgNP/Sol. A for 2 h and then challenged with bacteria. Each bar represents the mean and standard deviation of three independent experiments, 16 *larvae* were used in each condition. Asterisks denote statistically significant differences. ^**^p = 0.0041; ^***^p = 0.0003, and ^*^p = 0.0116 (ANOVA and RM ANOVA followed by *t*-test).

Alterations in total hemocyte density (THC) and differential hemocyte density (DHC) are factors that indicate the *larvae* response to bacterial infection as hemocytes play an important role in the fight against pathogens (Bergin et al., 2006; Coates et al., 2019). Though not all hemocytes are directly involved in phagocytosis, overall they act together to strengthen the immune response (Browne et al., 2013; Wojda, 2017). Hemocyte density was evaluated by counting the different cell types in a hemocytometer chamber under a bright field microscope. Figure 5A displays a representative micrograph containing the hemocyte types present in the *larvae*: plasmatocytes (Pl), granular cells (Gr), oenocytoids (Oe) and spherulocytes (Sp). Figure 5B shows the THC at 18 h following the beginning of infection. *larvae* infected with *P. aeruginosa* (bricked and chessboard-like bars in the figure) showed reduced levels of hemocytes when compared to the *larvae* prophylactly treated with AgNPs (PA14+AgNP/Sol. A, solid black bar) (*p* = 0.0041; ANOVA and Tukey's followed by *t*-test).

The differential hemocyte count (DHC) of circulating hemocytes showed that the phagocytic population (Pl/Gr) of *larvae* infected with *P. aeruginosa* but not treated with AgNPs was lower than in the AgNP-treated group (Figure 5C, compare bricked and chessboard-like bars with solid black bar, *p* = 0.0003, ANOVA and Tukey's followed by *t*-test). Although there were not statistically significant differences in the oenocytoid population across the experimental groups, the phenoloxidase activity and microscopy analysis revealed that the infected groups (PA14 and PA14 + Sol. A) displayed the highest level of black cell aggregates (**Figure 7B**) and the highest ProPo activity rate (Figure 5A—bricked and chessboard-like bars), both of which are directly related to an increment in oenocytoids Finally, the level of spherulocytes in the infected group pre-treated with AgNPs was higher than in the other groups (*p* = 0.0116; ANOVA and Tukey's followed by *t*-test) (Figure 5C). Though spherulocytes are apparently not directly involved in the insect immune response, these cells may contribute to *larvae* protection and survival by increasing their ability to scavenge nutrients (Wu et al., 2016; Wojda, 2017). It should be noted that *larvae* treated with Sol. A or AgNPs + Sol. A alone showed similar levels of THC when compared to the sham group – *larvae* that were not manipulated (data not shown), indicating that the AgNPs do not exert adverse effects on hemocytes.

### Induction of Phenoloxidase Activity

**Figure 6:**
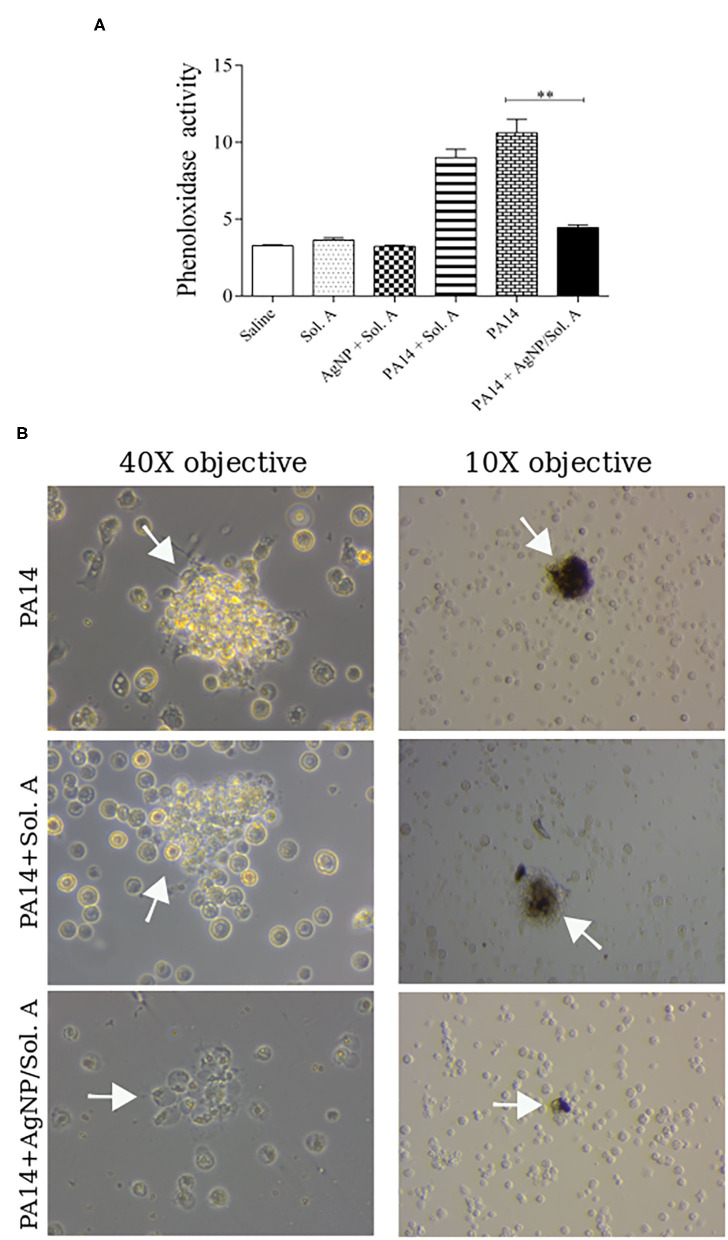
**(A)** Phenoloxidase activity at 9 h following infection with *P. aeruginosa* was determined by analyzing the rate of melanization at 0, 15, 30, 45, 60, 75, and 90 min. The bars representations are as described in the legend of Figure 4. Each bar represents the mean and standard deviations of three independent experiments. Each treatment condition contained 16 *larvae*. ^**^p = 0.0031 (ANOVA and RM ANOVA followed by *t*-test). **(B)** Microscope images showing cell aggregates on the left and black aggregates on the right (white arrows) resulting from PO activity.

In invertebrates, the first step in the immune response cascade is the activation of phenoloxidase (PO), which has a strong cytotoxic effect against non-self molecules (Wojda, 2017). The level of PO activation in *larvae* infected with*P. aeruginosa* and pre-treated with AgNPs was evaluated (Figure 6A). The hemolymph extracted from infected *larvae* displayed a high PO activity throughout the experiment. On the other hand, the group pre-treated with AgNPs showed a PO activity similar to that of the non-infected groups (compare PA14 and PA14 + Sol. A bars with PA14+AgNP/Sol. A solid black bar, *p* = 0.0031, ANOVA followed by *t*-test). Figure 6B displays representative pictures of PO within hemocytes synthesized in response to PA14 infection. The dark spots result from the activation of the PO cascade.

### Quantification of Cell Aggregates and Nodulation

**Figure 7:**
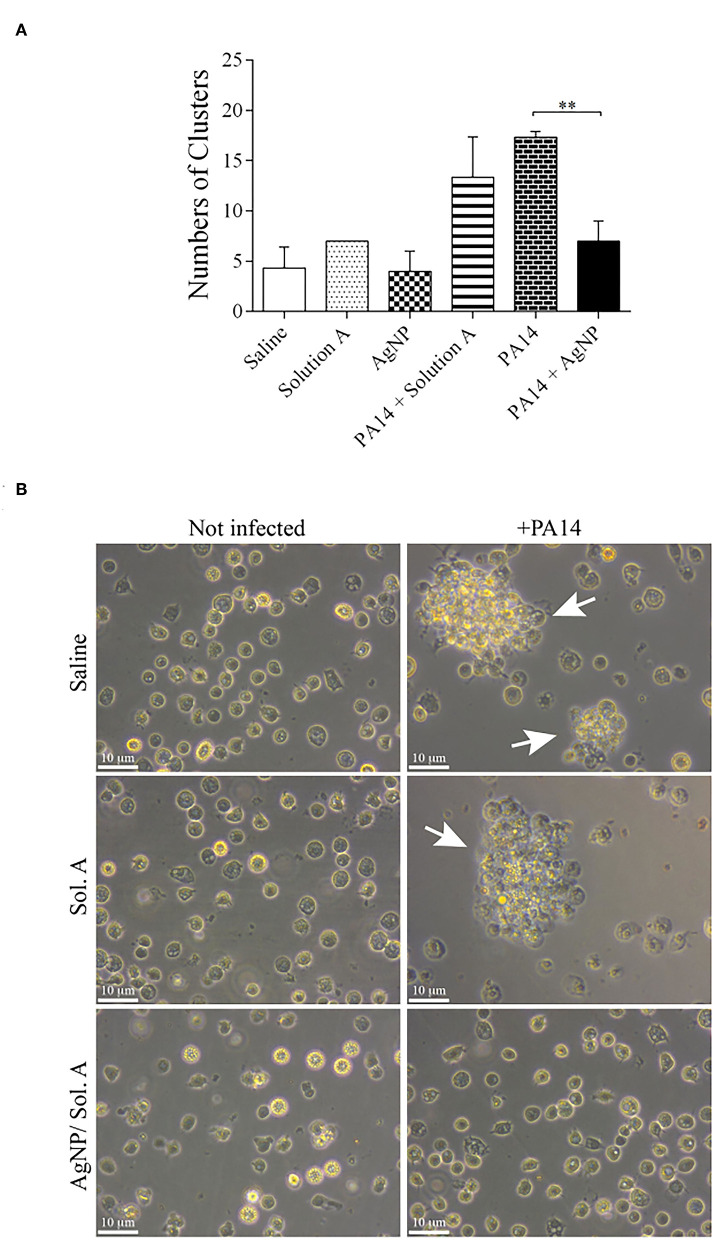
**(A)** Quantification of cell clusters during the nodulation process. The number of clusters was determined by counting cell aggregates (nodulation) under a light microscope. The bars representations are as described in the legend of Figure 4. Each bar represents the mean and standard deviations of three independent experiments. Each treatment condition contained 16 *larvae*. ^**^p = 0.0010 (ANOVA and RM ANOVA followed by *t*-test). **(B)** Representative microscope images of cell nodulation. Micrographs on the left show hemocytes extracted from non-infected *larvae* and micrographs on the right show hemocytes from *larvae* treated with PA14 bacteria. White arrows indicate aggregates of cells.

The formation of hemocyte clusters, that contain microorganisms and occurs during the *larvae* healing process was visually followed under a microscope. Figure 7A shows the quantification of cell clusters, each containing at least 8 cells. The number of clusters was significantly higher in the *larvae* infected with *P. aeruginosa* than in *larvae* pre-treated with AgNPs [compare PA14 (bricked bar) or PA14 + Solution A (stripped bar) with PA14+AgNP/Sol. A (solid black bar), *p* = 0.001, ANOVA followed by *t*-test]. Figure 7B shows representative images of cell clusters.

## Discussion

A major advantage in using insects as model organisms is that the innate immune system of these invertebrates is functionally similar to that of mammals (Browne et al., 2013). Accordingly, human pathogens display comparable levels of virulence and susceptibility when used to infect *G. mellonella larvae* or mammals (Desbois and Coote, 2012). It has been shown that 25 PA14 bacteria were sufficient to kill *G. mellonella* in 48 h (Jander et al., 2000; Hill et al., 2014). Similarly, in the present study, we showed that 100 CFU of strain PA14 were sufficient to kill all *larvae* in the first 24 h. Higher amounts of bacteria killed the *larvae* in less than 12 h (not shown). It has also been shown that the MICs of other antipseudomonal drugs were equivalent in *G. mellonella* and in human patients (Hill et al., 2014). Our formulation of AgNPs displayed a strong *in vitro* bactericidal effect against strain PA14, with a MIC of 2 μ g/ml. The MICs of AgNPs in *P. aeruginosa* thus far reported in the literature were: 1.06 - 4.25 μ/ml in strains PA14, AC12A, and DIN1 of *P. aeruginosa* (Pompilio et al., 2018); 2 μ/ml in an environmental *P. aeruginosa* (Kora and Arunachalam, 2011); 3.4–27 μ/ml in a clinical isolate of *P. aeruginosa* or in strain CCM3955 (Panáček et al., 2018); 8 μ g/ml (*P. aeruginosa* PAO1) (Radzig et al., 2013); 10 μ/ml in *P. aeruginosa* PA14 (da Silva et al., 2020); 14 μ g/ml (*P. aeruginosa* strain CCM 3955) (Guzman et al., 2012); 16 μ g /ml (*P. aeruginosa* ATCC 10145) Wypij et al. (2018); 8 mg/ml (*P. aeruginosa* clinical isolate) Nour El Din et al. (2016) and 2.48 μ g/ml (clinical isolates) (Liao et al., 2019). Thus only one out of 9 studies (Pompilio et al., 2018) have reported an AgNP preparation with a lower MIC than ours.

Here, the *in vivo* minimal AgNPs concentration required to protect *G. mellonella larvae* against *P. aeruginosa* was 25 mg/Kg. A prophylactic treatment using this concentration of AgNPs resulted in the survival of 80% of the infected *larvae*. The discrepancy between *in vitro* and *in vivo* MICs is normal and has been reported elsewhere (Pompilio et al., 2018). A basic difference between *in vivo* and *in vitro* tests is that in the former endogenous molecules may interfere with the tested compound activity. For instance, circulating albumin in mammals blood is known to interact with many different molecules, reducing their effectiveness (Yamasaki et al., 2013).

The immune response of insects is a multi-step process, activated upon pathogen invasion, that can be followed through the analyses of a variety of endpoints, such as *larvae* survival, hemocyte characterization and quantification, presence of nodulation and melanization, presence of antimicrobial peptides, ROS synthesis and lactate dehydrogenase activity (Rowan et al., 2009; Browne and Kavanagh, 2013). The cellular response is mediated by six different types of hemocytes, with three of them being the most relevant: granular cells, plasmatocytes and oenocytoids. Granular cells and plasmatocytes are adherent cells that possess the ability to carry out phagocytosis, encapsulation and nodulation of the pathogen. Though oenocytoids are also able to carry out phagocytosis (Wu et al., 2016), their main functions are the synthesis and transport of mucopolysaccharides and, most importantly, the secretion of serine protease associated with phenoloxidase (PO) activity (Tokura et al., 2014; Wojda, 2017). Given the crucial role that hemocytes play in the response to invading agents, the investigation of hemocyte build-up reveals important clues about the modulation of *G. mellonella* immune system. The proportion of each hemocyte type has been shown to vary widely during the 7 stages of larval development (Bergin et al., 2006). Pre-hemocytes have a short half-life, which explains their absence in this as well as in other studies (Labbé and Saleh, 2008; Dubovskiy et al., 2013). The distribution of different hemocyte types observed in the present study showed a considerable fluctuation but is consistent with what has been reported elsewhere (Bergin et al., 2006). We showed that the concentration of total hemocytes in *larvae* treated prophylactically with AgNPs was higher than in the non-treated *larvae*. The restoration of THC in the AgNP-treated *larvae* is likely to be associated with bacterial clearance from the *larvae* body. Similarly, Rowan et al. (2009) demonstrated that the administration of AgNO_3_ derivatives prior to infection with Candida albicans protected *G. mellonella*. The efficacy of these compounds were attributed to an increase in hemocyte counting, mostly of phagocytic cells.

It has been shown that *P. aeruginosa* infection in *G. mellonella* causes morphological and ultrastructural changes in hemocytes, including the formation of autophagic vacuoles, indicating that *P. aeruginosa* actively destroys hemocytes (Andrejko et al., 2014; Mizerska-Dudka and Andrejko, 2014). A similar effect was shown in this study, as our hemolymph microscopic analyses revealed the presence of phagocytic vacuoles and membrane alterations akin to the spreading of phagocytic vertebrate cells. The pathogen ability to invade the host is associated with virulence factors which ultimately elicit the insect cellular and humoral immune response. Plasmatocytes and granular cells are the main phagocytic cells that engulf and kill invading bacteria. These cells undergo cytoskeleton rearrangement, followed by extensive pseudopodia. The final step of this process is the phagocytosis of the invading pathogen or its envelopment in a nodular or capsular structure (Wojda and Jakubowicz, 2007; Mizerska-Dudka and Andrejko, 2014; Wojda, 2017). The microscope analyses revealed that prophylactic administration of AgNPs reduced or eliminated the presence of bacteria in the larva body resulting in less membrane disruption.

Our results suggest that the role of AgNPs on the protection against *P. aeruginosa* infection was double-fold: they actively killed the invading bacteria and indirectly act upon the *larvae* immune system, curbing an exacerbated immune response caused by the production of quinones and melanization that may eventually harm the host tissues. However, it is still possible that the alleviation of the immune response could be entirely due to the elimination of *P. aeruginosa* cells by the bactericidal activity of AgNPs.

In all tested groups hemocytes surrounded the *P. aeruginosa* cells, forming nodules. There was a positive correlation between the number of nodules and bacteria counts in the *larvae*. Accordingly, *larvae* pre-treated with AgNPs presented less bacteria and accumulated fewer nodules, corroborating the association between the absence of nodules and bacterial clearance (Hill et al., 2014). The cellular response occurs concomitantly to the humoral response, which is mediated by defense molecules, such as antimicrobial peptides, and oxygen and nitrogen free radicals (Rowan et al., 2009). In addition to these mechanisms, insects display a complex enzymatic cascade that results in melanization, a coagulation process that is characterized by the formation of capsules of dark composition that engulf the invading agent (Tokura et al., 2014). Prophylactic treatment with AgNPs treatment has also reduced the level of melanization and PO activity. This achievement is of utmost importance as high levels of melanization results in the death of both host and pathogen (Tokura et al., 2014).

Another type of hemocyte present in the nodules is the oenocytoid. The concentration of these cells was similar in the AgNP-treated group and in the non-infected controls. The oenocytoids are involved in the production of serine proteases that activate the phenoloxidase cascade, whose final product is melanin (Tokura et al., 2014). Low levels of these cells might explain the low phenoloxidase activity and the consequent reduction of clusters observed in PA14-infected *larvae* pre-treated with AgNPs.

Nanoparticles have many advantages over conventional antimicrobial agents, including antibiotics. They are usually inexpensive and are simple to prepare. The green synthesis with kefir used in this study simplifies and cheapen even more the process of AgNP synthesis. Due to their small dimensions, nanoparticles penetrate easily in the bacterial cell or in the complex matrix of biofilm communities and promptly kill the bacteria. In addition, silver nanoparticles are more potent than ionic silver, as the latter is usually inactivated by complexation and precipitation and in addition are cytotoxic to human cells (Kora and Arunachalam, 2011). Finally, due to the multiple and simultaneous mechanisms of AgNP action the pathogen is less likely to develop resistance against these particles (Klueh et al., 2000; Mathur et al., 2018; Pompilio et al., 2018). Even though the antimicrobial effect of AgNPs alone was quite remarkable, a combination with one or more antibiotics would considerably strengthen the treatment and would likely overcome bacterial resistance. Combinations of drugs, such as *beta*-lactams and aminoglycosides are already been used to treat *P. aeruginosa* infections in humans (Fritzenwanker et al., 2018).

The exact mechanism through which AgNPs kill bacteria is still unclear. Two different mechanisms have been proposed: killing by contact and Ag^+^-mediated death. In the former, a strong interaction between AgNPs and the peptidoglycan layer takes place, which eventually generates openings in the cell wall resulting in the extravasation of the cellular content (Sondi and Salopek-Sondi, 2004; Rai et al., 2012; Franci et al., 2015). Another mechanism suggests that AgNPs penetrate the microbial cell, where they interact with cellular structures and biomolecules, such as proteins, lipids, and DNA. The interaction of these macromolecules with the AgNPs leads to bacterial dysfunction and ultimately death. In particular, AgNPs interact with ribosomes resulting in their denaturation and consequently protein synthesis inhibition (Morones et al., 2005). AgNPs also have the ability to produce high levels of reactive oxygen species (ROS), such as hydrogen peroxide, anion superoxide, hydroxyl radical, hypochlorous acid and singlet oxygen (Kalishwaralal et al., 2010), that attack bacterial structures and macromolecules. The immune response of the *larvae* fight the remaining bacteria by synthesizing hemocytes (Mizerska-Dudka and Andrejko, 2014), antimicrobial peptides, lysozyme and by activating the PO cascade (Morones et al., 2005; Tokura et al., 2014; Wojda, 2017).

In conclusion, here, we presented for the first time a thorough analysis of the antipseudomonal effect of biogenic AgNPs on *G. mellonella larvae*. A prophylactic treatment with silver nanoparticles efficiently protected *G. mellonella* from bacterial infection by killing the invading bacteria and possibly by regulating the larva's immune response. Based on the analysis of 6 endpoints: survival assay, bacterial load, quantification and qualification of hemocytes, phenoloxidase activity and quantification of nodules, the present work validated the use of *G. mellonella* as a model for testing antipseudomonal drugs and for the study of bacterial pathogenesis and host response.

## Data Availability Statement

The raw data supporting the conclusions of this article will be made available by the authors, without undue reservation.

## Author Contributions

LT and LG contributed to this study, by designing and conducting all experiments, and helped drafting the manuscript. FS synthesized and analyzed the physico-chemical activities of silver nanoparticles. MC conducted the microscopic analyses. CT gave support and helped drafting the manuscript. BS coordinated the study and drafted the manuscript. All authors contributed to the article and approved the submitted version.

## Conflict of Interest

The authors declare that the research was conducted in the absence of any commercial or financial relationships that could be construed as a potential conflict of interest.

## References

[B1] Andrejko M. Zdybicka-Barabas A. Cytryńska M. (2014). Diverse effects of *Galleria mellonella* infection with entomopathogenic and clinical strains of *Pseudomonas aeruginosa*. J. Invertebr. Pathol. 115, 14–25. 10.1016/j.jip.2013.10.006 24513029

[B2] Banville N. Browne N. Kavanagh K. (2012). Effect of nutrient deprivation on the susceptibility of *Galleria mellonella* larvae to infection. Virulence 3, 497–503. 10.4161/viru.21972 23076277 PMC3524148

[B3] Beeton M. Alves D. Enright M. Jenkins A. (2015). Assessing phage therapy against *Pseudomonas aeruginosa* using a *Galleria mellonella* infection model. Int. J. Antimicrob. Agents 46, 196–200. 10.1016/j.ijantimicag.2015.04.005 26100212

[B4] Bergin D. Murphy L. Keenan J. Clynes M. Kavanagh K. (2006). Pre-exposure to yeast protects larvae of *Galleria mellonella* from a subsequent lethal infection by *Candida albicans* and is mediated by the increased expression of antimicrobial peptides. Microbes Infect. 8, 2105–2112. 10.1016/j.micinf.2006.03.005 16782387

[B5] Browne N. Heelan M. Kavanagh K. (2013). An analysis of the structural and functional similarities of insect hemocytes and mammalian phagocytes. Virulence 4, 597–603. 10.4161/viru.25906 23921374 PMC3906293

[B6] Browne N. Kavanagh K. (2013). Developing the potential of using *Galleria mellonella* larvae as models for studying brain infection by *Listeria monocytogenes*. Virulence 4, 271–2. 10.4161/viru.24174 23552811 PMC3710329

[B7] Coates C. J. Lim J. Harman K. Rowley A. F. Griffiths D. J. Emery H.. (2019). The insect, *Galleria mellonella*, is a compatible model for evaluating the toxicology of okadaic acid. Cell Biol. Toxicol. 35, 219–232. 10.1007/s10565-018-09448-2 30426330 PMC6556153

[B8] Cutuli M. A. Petronio G. Vergalito F. Magnifico I. Pietrangelo L. Venditti N.. (2019). *Galleria mellonella* as a consolidated *in vivo* model hosts: new developments in antibacterial strategies and novel drug testing. Virulence 10, 527–541. 10.1080/21505594.2019.1621649 31142220 PMC6550544

[B9] da Silva R. T. P. Petri M. V. Valencia E. Y. Camargo P. H. C. de Torresi S. I. C. Spira B. (2020). Visible light plasmon excitation of silver nanoparticles against antibiotic-resistant *Pseudomonas aeruginosa*. Photodiagn. Photodyn. Therapy 31:101908. 10.1016/j.pdpdt.2020.101908 32634655

[B10] Decraene V. Ghebrehewet S. Dardamissis E. Huyton R. Mortimer K. Wilkinson D.. (2018). An outbreak of multidrug-resistant *Pseudomonas aeruginosa* in a burns service in the North of England: challenges of infection prevention and control in a complex setting. J. Hosp. Infect. 100, e239–e245. 10.1016/j.jhin.2018.07.012 30012376

[B11] Desbois A. P. Coote P. J. (2012). Utility of greater wax moth larva (*Galleria mellonella*) for evaluating the toxicity and efficacy of new antimicrobial agents. Adv. Appl. Microbiol. 78, 25–53. 10.1016/B978-0-12-394805-2.00002-6 22305092

[B12] Dubovskiy I. M. Whitten M. M. A. Yaroslavtseva O. N. Greig C. Kryukov V. Y. Grizanova E. V.. (2013). Can insects develop resistance to insect pathogenic fungi? PLoS ONE 8:e60248. 10.1371/journal.pone.0060248 23560083 PMC3613352

[B13] Emerson J. Rosenfeld M. McNamara S. Ramsey B. Gibson R. L. (2002). *Pseudomonas aeruginosa* and other predictors of mortality and morbidity in young children with cystic fibrosis. Pediatr. Pulmonol. 34, 91–100. 10.1002/ppul.10127 12112774

[B14] Fox C. L. (1968). Silver sulfadiazine-a new topical therapy for *Pseudomonas* in burns. Arch. Surg. 96:184. 10.1001/archsurg.1968.01330200022004 5638080

[B15] Franci G. Falanga A. Galdiero S. Palomba L. Rai M. Morelli G.. (2015). Silver nanoparticles as potential antibacterial agents. Molecules 20, 8856–8874. 10.3390/molecules20058856 25993417 PMC6272636

[B16] Fritzenwanker M. Imirzalioglu C. Herold S. Wagenlehner F. M. Zimmer K.-P. Chakraborty T. (2018). Treatment options for carbapenem- resistant gram-negative infections. Deutsch. Arzte. Inter. 115, 345–352. 10.3238/arztebl.2018.0345 29914612 PMC6172649

[B17] Guzman M. Dille J. Godet S. (2012). Synthesis and antibacterial activity of silver nanoparticles against gram-positive and gram-negative bacteria. Nanomed. Nanotechnol. Biol. Med. 8, 37–45. 10.1016/j.nano.2011.05.007 21703988

[B18] He J. Baldini R. L. Daziel E. Saucier M. Zhang Q. Liberati N. T.. (2004). The broad host range pathogen *Pseudomonas aeruginosa* strain pa14 carries two pathogenicity islands harboring plant and animal virulence genes. Proc. Natl. Acad. Sci. U.S.A. 101, 2530–2535. 10.1073/pnas.0304622101 14983043 PMC356984

[B19] Hill L. Veli N. Coote P. J. (2014). Evaluation of *Galleria mellonella* larvae for measuring the efficacy and pharmacokinetics of antibiotic therapies against *Pseudomonas aeruginosa* infection. Int. J. Antimicrob. Agents 43, 254–61. 10.1016/j.ijantimicag.2013.11.001 24361354

[B20] Jain J. Arora S. Rajwade J. M. Omray P. Khandelwal S. Paknikar K. M. (2009). Silver nanoparticles in therapeutics: development of an antimicrobial gel formulation for topical use. Mol. Pharmaceutics 6, 1388–1401. 10.1021/mp900056g 19473014

[B21] Jander G. Rahme L. G. Ausubel F. M. (2000). Positive correlation between virulence of *Pseudomonas aeruginosa* mutants in mice and insects. J. Bacteriol. 182, 3843–3845. 10.1128/JB.182.13.3843-3845.2000 10851003 PMC94559

[B22] Kalishwaralal K. BarathManiKanth S. Pandian S. R. K. Deepak V. Gurunathan S. (2010). Silver nanoparticles impede the biofilm formation by *Pseudomonas aeruginosa* and *Staphylococcus epidermidis*. Coll. Surf. B Biointerfaces 79, 340–4. 10.1016/j.colsurfb.2010.04.014 20493674

[B23] Karaiskos I. Antoniadou A. Giamarellou H. (2017). Combination therapy for extensively-drug resistant gram-negative bacteria. Expert Rev. Anti Infect. Ther. 15, 1123–1140. 10.1080/14787210.2017.1410434 29172792

[B24] Klueh U. Wagner V. Kelly S. Johnson A. Bryers J. D. (2000). Efficacy of silver-coated fabric to prevent bacterial colonization and subsequent device-based biofilm formation. J. Biomed. Mater. Res. 53, 621–631. 10.1002/1097-4636(2000)53:6<621::AID-JBM2>3.0.CO;2-Q 11074419

[B25] Kora A. J. Arunachalam J. (2011). Assessment of antibacterial activity of silver nanoparticles on *Pseudomonas aeruginosa* and its mechanism of action. World J. Microbiol. Biotechnol. 27, 1209–1216. 10.1007/s11274-010-0569-2

[B26] Labbé K. Saleh M. (2008). Cell death in the host response to infection. Cell Death Diff. 15, 1339–1349. 10.1038/cdd.2008.91 18566602

[B27] Laureys D. De Vuyst L. (2014). Microbial species diversity, community dynamics, and metabolite kinetics of water kefir fermentation. Appl. Environ. Microbiol. 80, 2564–72. 10.1128/AEM.03978-13 24532061 PMC3993195

[B28] Lee D. G. Urbach J. M. Wu G. Liberati N. T. Feinbaum R. L. Miyata S.. (2006). Genomic analysis reveals that *Pseudomonas aeruginosa* virulence is combinatorial. Genome Biol. 7:R90. 10.1186/gb-2006-7-10-r90 17038190 PMC1794565

[B29] Liao S. Zhang Y. Pan X. Zhu F. Jiang C. Liu Q.. (2019). Antibacterial activity and mechanism of silver nanoparticles against multidrug-resistant. Int. J. Nanomed. 14, 1469–1487. 10.2147/IJN.S191340 30880959 PMC6396885

[B30] Magiorakos A.-P. Srinivasan A. Carey R. B. Carmeli Y. Falagas M. E. Giske C. G.. (2012). Multidrug-resistant, extensively drug-resistant and pandrug-resistant bacteria: an international expert proposal for interim standard definitions for acquired resistance. Clin. Microbiol. Infect. 18, 268–81. 10.1111/j.1469-0691.2011.03570.x 21793988

[B31] Markowska K. Grudniak A. M. Wolska K. I. (2013). Silver nanoparticles as an alternative strategy against bacterial biofilms. Acta Biochim. Pol. 60, 523–30. 10.18388/abp.2013_2016 24432308

[B32] Mathee K. (2018). Forensic investigation into the origin of pseudomonas aeruginosa pa14 - old but not lost. J. Med. Microbiol. 67, 1019–1021. 10.1099/jmm.0.000778 30067168

[B33] Mathur P. Jha S. Ramteke S. Jain N. K. (2018). Pharmaceutical aspects of silver nanoparticles. Art. Cells Nanomed. Biotech. 46(Suppl 1):115–126. 10.1080/21691401.2017.1414825 29231755

[B34] Miller J. H. (1992). A Short Course in Bacterial Genetics: A Laboratory Manual and Handbook for Escherichia coli and Related Bacteria. Cold Spring Harbor, NY: Cold Spring Harbor Laboratory.

[B35] Miyata S. Casey M. Frank D. W. Ausubel F. M. Drenkard E. (2003). Use of the *Galleria mellonella* caterpillar as a model host to study the role of the type III secretion system in *Pseudomonas aeruginosa* pathogenesis. Infect. Immun. 71, 2404–2413. 10.1128/IAI.71.5.2404-2413.2003 12704110 PMC153283

[B36] Mizerska-Dudka M. Andrejko M. (2014). Galleria mellonella hemocytes destruction after infection with *Pseudomonas aeruginosa. J. Basic Microbiol*. 54, 232–46. 10.1002/jobm.201200273 23456635

[B37] Morones J. R. Elechiguerra J. L. Camacho A. Holt K. Kouri J. B. Ramírez J. T.. (2005). The bactericidal effect of silver nanoparticles. Nanotechnology 16, 2346–53. 10.1088/0957-4484/16/10/059 20818017

[B38] Nour El Din S. El-Tayeb T. A. Abou-Aisha K. El-Azizi M. (2016). In vitro and *in vivo* antimicrobial activity of combined therapy of silver nanoparticles and visible blue light against *Pseudomonas aeruginosa. Int. J. Nanomed*. 11, 1749–1758. 10.2147/IJN.S102398 27175075 PMC4854264

[B39] Panáček A. Kvítek L. Smékalová M. Večerová R. Kolár M. Röderová M.. (2018). Bacterial resistance to silver nanoparticles and how to overcome it. Nat. Nanotechnol. 13, 65–71. 10.1038/s41565-017-0013-y 29203912

[B40] Pompilio A. Geminiani C. Bosco D. Rana R. Aceto A. Bucciarelli T.. (2018). Electrochemically synthesized silver nanoparticles are active against planktonic and biofilm cells of *Pseudomonas aeruginosa* and other cystic fibrosis-associated bacterial pathogens. Front. Microbiol. 9:1349. 10.3389/fmicb.2018.01349 30026732 PMC6041389

[B41] Potron A. Poirel L. Nordmann P. (2015). Emerging broad-spectrum resistance in *Pseudomonas aeruginosa* and *Acinetobacter baumannii*: mechanisms and epidemiology. Int. J. Antimicrob. Agents 45, 568–85. 10.1016/j.ijantimicag.2015.03.001 25857949

[B42] Radzig M. Nadtochenko V. Koksharova O. Kiwi J. Lipasova V. Khmel I. (2013). Antibacterial effects of silver nanoparticles on gram-negative bacteria: Influence on the growth and biofilms formation, mechanisms of action. Colloids Surf. B 102, 300–306. 10.1016/j.colsurfb.2012.07.039 23006569

[B43] Rai M. Deshmukh S. Ingle A. Gade A. (2012). Silver nanoparticles: the powerful nanoweapon against multidrug-resistant bacteria. J. Appl. Microbiol. 112, 841–852. 10.1111/j.1365-2672.2012.05253.x 22324439

[B44] Rodríguez-Rojas A. Oliver A. Blázquez J. (2012). Intrinsic and environmental mutagenesis drive diversification and persistence of *Pseudomonas aeruginosa* in chronic lung infections. J. Infect. Dis. 205, 121–7. 10.1093/infdis/jir690 22080096

[B45] Rowan R. Moran C. McCann M. Kavanagh K. (2009). Use of *Galleria mellonella* larvae to evaluate the *in vivo* anti-fungal activity of [ag 2 (mal)(phen) 3]. Biometals 22:461. 10.1007/s10534-008-9182-3 19082779

[B46] Schneider C. A. Rasband W. S. Eliceiri K. W. (2012). NIH Image to ImageJ: 25 years of image analysis. Nat. Methods 9, 671–5. 10.1038/nmeth.2089 22930834 PMC5554542

[B47] Shrivastava S. Bera T. Roy A. Singh G. Ramachandrarao P. Dash D. (2007). Characterization of enhanced antibacterial effects of novel silver nanoparticles. Nanotechnology 18:225103 10.1088/0957-4484/18/22/225103 37016550

[B48] Singh B. R. Singh B. N. Singh A. Khan W. Naqvi A. H. Singh H. B. (2015). Mycofabricated biosilver nanoparticles interrupt *Pseudomonas aeruginosa* quorum sensing systems. Sci. Rep. 5:13719. 10.1038/srep13719 26347993 PMC4562228

[B49] Sondi I. Salopek-Sondi B. (2004). Silver nanoparticles as antimicrobial agent: a case study on *E. coli* as a model for Gram-negative bacteria. J. Colloid Interface Sci. 275, 177–82. 10.1016/j.jcis.2004.02.012 15158396

[B50] Tokura A. Fu G. S. Sakamoto M. Endo H. Tanaka S. Kikuta S.. (2014). Factors functioning in nodule melanization of insects and their mechanisms of accumulation in nodules. J. Insect Physiol. 60, 40–9. 10.1016/j.jinsphys.2013.11.003 24262307

[B51] Wiehlmann L. Wagner G. Cramer N. Siebert B. Gudowius P. Morales G.. (2007). Population structure of *Pseudomonas aeruginosa*. Proc. Natl. Acad. Sci. U.S.A. 104, 8101–8106. 10.1073/pnas.0609213104 17468398 PMC1876578

[B52] Wojda I. (2017). Immunity of the greater wax moth *Galleria mellonella*. Insect Sci. 24, 342–357. 10.1111/1744-7917.12325 26847724

[B53] Wojda I. Jakubowicz T. (2007). Humoral immune response upon mild heat-shock conditions in *Galleria mellonella* larvae. J. Insect Physiol. 53, 1134–44. 10.1016/j.jinsphys.2007.06.003 17631308

[B54] Wu D. Fan W. Kishen A. Gutmann J. L. Fan B. (2014). Evaluation of the antibacterial efficacy of silver nanoparticles against *Enterococcus faecalis* biofilm. J. Endod. 40, 285–290. 10.1016/j.joen.2013.08.022 24461420

[B55] Wu G. Liu Y. Ding Y. Yi Y. (2016). Ultrastructural and functional characterization of circulating hemocytes from *Galleria mellonella* larva: cell types and their role in the innate immunity. Tissue Cell 48, 297–304. 10.1016/j.tice.2016.06.007 27378036

[B56] Wypij M. Czarnecka J. Świecimska M. Dahm H. Rai M. Golinska P. (2018). Synthesis, characterization and evaluation of antimicrobial and cytotoxic activities of biogenic silver nanoparticles synthesized from *Streptomyces xinghaiensis* OF1 strain. World J. Microbiol. Biotechnol. 34:23. 10.1007/s11274-017-2406-3 29305718 PMC5756267

[B57] Yamasaki K. Chuang V. T. G. Maruyama T. Otagiri M. (2013). Albumin-drug interaction and its clinical implication. Biochim. Biophys. Acta Gen. Subjects 1830, 5435–5443. 10.1016/j.bbagen.2013.05.005 23665585

[B58] Yan X. He B. Liu L. Qu G. Shi J. Hu L.. (2018). Antibacterial mechanism of silver nanoparticles in *Pseudomonas aeruginosa*: proteomics approach. Metallomics 10, 557–564. 10.1039/C7MT00328E 29637212

